# Genomic characterisation of respiratory syncytial virus: a novel system for whole genome sequencing and full-length G and F gene sequences

**DOI:** 10.2807/1560-7917.ES.2023.28.49.2300637

**Published:** 2023-12-07

**Authors:** María Iglesias-Caballero, Sara Camarero-Serrano, Sarai Varona, Vicente Mas, Cristina Calvo, María Luz García, Juan García-Costa, Sonia Vázquez-Morón, Sara Monzón, Albert Campoy, Isabel Cuesta, Francisco Pozo, Inmaculada Casas

**Affiliations:** 1 Laboratory of Reference and Research in Respiratory Viruses, National Centre for Microbiology, Instituto de Salud Carlos III, Majadahonda, Spain; 2 Bioinformatics Unit, Unidades Centrales Científico Técnicas, Instituto de Salud Carlos III, Majadahonda, Spain; 3 Paediatric Infectious and Tropical Diseases Department, Hospital Universitario La Paz, Hospital La Paz Institute for Health Research (IdiPAZ Foundation), Madrid, Spain; 4 CIBER de Enfermedades Infecciosas (CIBERINFEC), ISCIII, Madrid, Spain; 5 CIBER de Epidemiología y Salud Pública (CIBERESP), ISCIII, Madrid, Spain; 6 Paediatric Department, Severo Ochoa University Hospital, Leganés, Biomedical Sciences Research Institute, Puerta de Hierro-Majadahonda University Hospital, Madrid, Spain; 7 Santa María Nai Hospital, Ourense, Spain; * These authors contributed equally

**Keywords:** Respiratory Syncytial virus, whole genome sequencing, surveillance

## Abstract

To advance our understanding of respiratory syncytial virus (RSV) impact through genomic surveillance, we describe two PCR-based sequencing systems, (i) RSVAB-WGS for generic whole-genome sequencing and (ii) RSVAB-GF, which targets major viral antigens, G and F, and is used as a complement for challenging cases with low viral load. These methods monitor RSV genetic diversity to inform molecular epidemiology, vaccine effectiveness and treatment strategies, contributing also to the standardisation of surveillance in a new era of vaccines.

At present, several RSV vaccine candidates are approved, pending regulatory body approval or in the final stages of clinical trials [1]. In addition to immunisations, the use of the monoclonal antibodies, palivizumab [2] and nirsevimab [3], is recommended to prevent RSV infection in infants and available in several countries [4]. The need to develop methods for monitoring the impact of monoclonal antibodies and vaccine effectiveness is becoming more urgent for the national and supranational surveillance systems.

In this study, we propose two methods for characterising respiratory syncytial virus (RSV) subtypes A and B: RSVAB-WGS, a novel amplicon-based system for RSV whole genome sequencing (WGS) and RSVAB-GF, a method focused on obtaining the specific sequences of the main antigens, glycoprotein (G) and fusion protein (F). 

## Design and evaluation of RSVAB-WGS targeted PCR

For the design, we aligned 922 selected sequences obtained from the GenBank and GISAID databases. We chose a total of 12 primers for the RSVAB-WGS method and two additional primers for RSVAB-GF method (Table 1). A mix of these primers collectively cover both RSV subtypes, generating PCR fragments of an average size of 1.5–2.5 kb in the WGS and 3.0–3.5 kb in the GF protocol. Both amplification protocols are openly available on the platform *protocols.io* [5]. 

**Table 1:** Primers used for the respiratory syncytial virus RSVAB-WGS and RSVAB-GF sequencing method

Primer	Sequence (5’–3’)
Mix 1 RSVAB WGS	
RSVCombinitial	ACGCGAAAAAATGCGTACWACA
RSVWGS4R	CATGWTGWYTTATTTGCCCC
RSVWGS2F	CACTWACAATATGGGTGCC
RSVWGS1R	TCCATKGTTATTTGCCCC
RSVWGS3.2F	ACATGGAAAGAYATYAGCC
RSVWGS2R	CRTTYCTTAARGTRGGCC
RSVWGS3.2R	TTGCATCTGTAGCAGGAATGG
RSVCombending	ACGAGAAAAAAAGTGTCAAAAACTAA
Mix 2 RSVAB WGS	
RSVCombinitial	ACGCGAAAAAATGCGTACWACA
RSVWGS1R	TCCATKGTTATTTGCCCC
RSVWGS2F	CACTWACAATATGGGTGCC
RSVWGS8R	TCMAWYTCWGCAGCTCC
RSVWGS5R	CAAACATTTAATCTRCTAAGGC
RSVWGS6F	TTATAYAGATATCAYATGGGTGG
RSVWGS6R	CCCTCTCCCCAATCTTTTTC
RSVCombending	ACGAGAAAAAAAGTGTCAAAAACTAA
Mix RSVAB-GF	
OG1-21	GGGGCAAATGCAACCATGTCC
RSVGF-R	TTCGYGACATATTTGCCCC

The primers were organised by their position in the genome and the amplification mix in which they were employed. Primers RSVCombinitial, RSVWGS1R, RSVWGS2F and RSVCombending were used in both mixes.

The PCR parameters and the final sensitivity of the chosen protocol were evaluated using dilutions of well-characterised reference viruses, RSV A Long and RSV B CH-18537, as control samples (Table 2). Our method exhibited a sensitivity of 20 copies/mL for RSV A and 200 copies/mL for RSV B (Table 2).

**Table 2:** Respiratory syncytial virus reference controls used to standardise the RSVAB-WGS and RSVAB-GF sequencing methods

Strain	ATCC nomenclature	Cq value				Number of copies/mL			
Dilution of control virus		10^-1^	10^-2^	10^-3^	10^-4^	10^-1^	10^-2^	10^-3^	10^-4^
RSV A Long	VR-26	15.12	18.88	22.09	25.57	2 × 10^4^	2 × 10^3^	2 × 10^2^	2 × 10^1^
RSV B CH-18537	VR-1580	16.21	19.5	22.8	26.2	2 × 10^4^	2 × 10^3^	2 × 10^2^	NA

ATCC: American type culture collection; Cq: quantification cycle; NA: not applicable.

The standardisation process included quantifying the dilutions of each control virus based on the Cq value obtained in the RT-PCR used for viral detection and subtyping. Subsequently, we calculated the number of copies of viral nucleic acids in each dilution of the control viruses.

We constructed libraries following the instructions provided by the Illumina DNA library preparation kit (Illumina, United States) for 300-cycle cartridges in Illumina MiSeq and NextSeq sequencers. Sequences were analysed for viral genome reconstruction using the viralrecon pipeline v2.6.0. (https://github.com/nf-core/viralrecon) [6], implemented in Nextflow (https://www.nextflow.io). Phylogenetic analysis was conducted using FasTreeMP software [7] with a generalised time reversible (GTR) model and a bootstrap test of 1,000 iterations.

For validation, we analysed a total of 142 nasopharyngeal exudates (NPE) and nasopharyngeal aspirates (NPA) collected from RSV-positive patients during the epidemic seasons 2018/19, 2019/20 and 2021/22. To assess the RSV viral load and facilitate subsequent sequencing, we performed a real-time PCR to detect RSV and used the resulting quantification cycle (Cq) value as a preliminary indicator [1,8]. Despite the usefulness of setting a cut-off in the Cq values used to ensure the complete genome acquisition, in this work, a deliberate decision was made not to set it. The aim was to determine the limit of detection in the method by sequencing both our lowest Cq value sample from the season (14.33) and the highest (29.4).

## Complete genome sequencing of respiratory syncytial virus A and B

For surveillance purposes, the RSVAB-WGS method exhibited practical versatility and proved to be applicable to specimens of both RSV subtypes across a spectrum of viral loads. Validation involved analysing 34 positive clinical specimens, 16 RSV A and 18 RSV B, previously typed [8]. These samples showed Cq values ranging from 15 to 20 (10 RSV A, eight RSV B), 21 to 26 (six samples of either subtype) and exceeding 27 (four RSV B). For samples with Cq values ≤ 25, RSVAB-WGS achieved more than 90% genome coverage. For Cq values 26–27, coverage ranged from 60% to 90%, and for Cq values exceeding 27, coverage dropped to 50%. We append in Supplementary Table S1 the detailed sequencing parameters for RSV clinical specimens used in RSVAB WGS method including a description of subtypes, Cq values, sequencing/assembly metrics and GISAID accession numbers.

Recognising the importance of accurate RSV genomic characterisation, we developed the RSVAB-GF method to complement the genomic coverage for both major antigenic proteins in cases where WGS encountered coverage difficulties, such as in samples with low viral load or when the resources for sequencing were constrained. This method offers a simpler and more cost-effective approach to obtaining sequences of both antigens. Validation involved analysing 108 clinical specimens (74 RSV A, 34 RSV B) with Cq values ranging from 14.3 to 29.4 and in all cases, both genes were completely covered. We provide in Supplementary Table S2 a description of the samples used in the targeted RSV AB-GF method including sequence identification, subtype, Cq value and GISAID accession numbers.

## Genetic analysis of the genomes obtained with RSVAB-WGS and RSVAB-GF

To compare taxonomic classifications obtained from full genome sequences and partial G and F sequences, we used 14 sequences (seven RSV A, seven RSV B) collected during the 2021/22 season (Table 3). We used the whole genome to assign RSV clades because either the complete sequence or the F gene sequence was available to us in addition to the G gene sequence. These clade definitions were based on the proposed nomenclatures by Goya et al. [9] and Ramaekers et al. [10] using Nextstrain platform*.* Analysis of the 14 RSV A sequences with both methods demonstrated complete agreement in clade assignation. For the seven RSV A sequences, identified subclades included A.D.1, A.D.3 and A.D.5.2. Taxonomic assignments from seasons 2018/19 and 2019/20 showed the presence of subclades A.D.1, A.D.3 (both also circulating in the 2021/22 season) and subclade A.D.2.3 (Table 3). In the RSV B subtype, clade assignment revealed total agreement in taxonomic classification. The seven sequences from the 2021/22 season were identified as subclade B.D.5.2.1.1 with both methods.

**Table 3:** Comparison of the taxonomic classification and clades obtained from respiratory syncytial virus full genome sequences and G and F partial gene sequences

RSV sequence	Clade (RSVAB-WGS)	Clade (RSVAB-GF)
hRSV/A/Spain/MD-224273/2022	A.D.1	A.D.1
hRSV/A/Spain/MD-224532/2022	A.D.5.2	A.D.5.2
hRSV/A/Spain/MD-224512/2022	A.D.5.2	A.D.5.2
hRSV/A/Spain/MD-224870/2022	A.D.3.1	A.D.3.1
hRSV/A/Spain/MD-224747/2022	A.D.3	A.D.3
hRSV/A/Spain/MD-224741/2022	A.D.5.2	A.D.5.2
hRSV/A/Spain/MD-224199/2022	A.D.3	A.D.3
hRSV/B/Spain/MD-220011/2021	B.D.5.2.1.1	B.D.5.2.1.1
hRSV/B/Spain/MD-220471/2022	B.D.5.2.1.1	B.D.5.2.1.1
hRSV/B/Spain/MD-220289/2022	B.D.5.2.1.1	B.D.5.2.1.1
hRSV/B/Spain/MD-224976/2022	B.D.5.2.1.1	B.D.5.2.1.1
hRSV/B/Spain/MD-223807/2022	B.D.5.2.1.1	B.D.5.2.1.1
hRSV/B/Spain/MD-224877/2022	B.D.5.2.1.1	B.D.5.2.1.1
hRSV/B/Spain/MD-224510/2022	B.D.5.2.1.1	B.D.5.2.1.1

F: fusion protein; G: glycoprotein; RSV: respiratory syncytial virus; WGS: whole genome sequencing.

For the phylogenetic analysis (Figure), we selected sequences from the main antigens G and F due to the high number of sequences with good quality and coverage in the GISAID database in comparison with those of the complete genome. We constructed a separate tree for each RSV subtype. In both subtypes, sequence clustering was primarily determined by mutations spanning consecutive seasons rather than by year or geographical location. The phylogenetic analysis of 2,314 RSV A sequences, including 74 sequences from this study, and 2,875 RSV B sequences, with the 34 G and F sequences obtained from this work, corroborated previous taxonomic results (Figure) [11].

**Figure:**
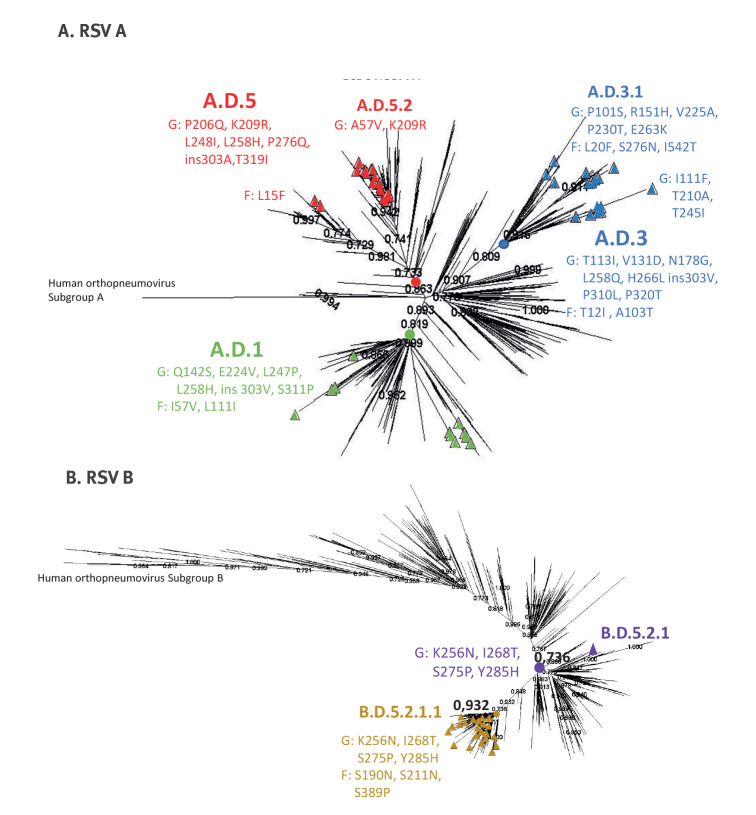
Phylogenetic tree of respiratory syncytial virus G and F sequences published in GISAID (n = 2,314 RSV A; n = 2,875 RSV B)

F: fusion protein; G: glycoprotein; RSV: respiratory syncytial virus.

The numbers represent percentage bootstrap support. The sequences obtained in this work were represented by triangles. Clusters, where these sequences were located, are identified by three colours (red, blue and green) according to the defining mutations. The colours yellow and purple mark the nodes with the defining mutations of the group.

## Discussion

Several methods have already been designed for sequencing RSV isolates or samples, employing diverse approaches [12-16]. However, the primary challenges in successful RSV sequencing often involve obtaining complete genomes from clinical samples with low viral loads [17] and high diversity of RNA genomes [18]. Previous methods often required prior subtyping and quantification due to differences in primer design based on subtypes [13,19], making the process expensive and labour-intensive. PCR amplicon sequencing, using amplicons generated through specific primers [20,21] or sequence-independent single primer amplification (SISPA) [13], exhibits notable advantages, particularly in clinical specimens with low viral loads [13]. The effectiveness of double-stranded cDNA generation before targeted amplification has proven effective for other respiratory viruses like severe acute respiratory syndrome coronavirus 2 (SARS-CoV-2) [22,23], and it was also validated in this work for RSV. The use of cDNA in our RSVAB- WGS method provided an augmented amount of viral nucleic acids using only a few microlitres of the clinical sample extract, optimising sample usage. Amplicon size is critical, as shorter amplicons can introduce biases in genome reconstruction and prevent the successful assembly of a full genome sequence for unknown viruses. The method described in this work produces amplicons compatible with both long and short-read sequencers, offering a versatile solution for laboratories, irrespective of the type of sequencer available.

To demonstrate the utility of our RSVAB-WGS and RSVAB-GF methods in molecular epidemiology studies of RSV, we conducted phylogenetic and genomic analyses using samples collected during the 2018/19, 2019/20 and 2021/22 epidemic RSV seasons. The phylogenetic analysis revealed that changes in the G protein gene had a larger impact on grouping than the timing of virus collection or geographical location. While WGS for monitoring viral transmission chains can offer great accuracy, studies indicate that the relationships identified with the G region are similar to the patterns observed with full-genome sequences [16]. Furthermore, a previous world-wide analysis of the RSV clades [11] demonstrated that entirely sequenced complete G gene sequences yielded a phylogenetic tree topology comparable to that obtained using whole genomes. This underscores the utility of the RSVAB-GF method in providing evolutionary information through a simplified system that could be used when the viral load is low and also when the resources for sequencing are not enough to support a WGS system.

In addition, we currently find it highly pertinent to characterise the F protein gene variability with an accurate and robust complementary method such as the RSVAB-GF PCR. This is especially crucial since the F protein is the main target for most vaccines, which are either already approved [1] or soon expected to be. Moreover, there are several monoclonal antibodies for RSV prevention [3], such as nirsevimab or palivizumab, all of which also target the F protein. 

Despite the advantages presented by these methods, we also have to address limitations in the WGS method, such as a decrease in coverage starting from Cq = 25 and the need to improve the complete genome sequencing in RSV B. Currently, the method is being improved to solve these issues. Although this work was confined to the sequences received in our laboratory, we believe this does not restrict the capability of these methods to sequence any type of RSV because the primer design was performed using sequences from various locations and different seasons.

The World Health Organization has started a global effort to homogenise RSV surveillance, based on the Global Influenza Surveillance and Response System [11]. The surveillance system needs to be supported by reliable and practical methods.

## Conclusions

While implementing WGS of RSV remains technically demanding and expensive, many laboratories express the need for guidance. Our universal amplicon-based system RSVAB-WGS is an accurate and cost-effective means to obtain genomic data. Coupled with the RSVAB-GF method, it enhances capacity and performance for high-quality sequence acquisition from both genes in a single run, even when processing hundreds of samples. These methods to characterise the complete RSV genome and the variability of G and F protein genes can serve as a useful tool for RSV surveillance, including monitoring of preventive measures to limit the spread of the virus.

## Supplementary Data

199000 Supplement

## References

[1] Venkatesan P. First RSV vaccine approvals. Lancet Microbe. 2023;4(8):e577. 10.1016/S2666-5247(23)00195-7 37390835

[2] Zhu Q McLellan JS Kallewaard NL Ulbrandt ND Palaszynski S Zhang J A highly potent extended half-life antibody as a potential RSV vaccine surrogate for all infants. Sci Transl Med. 2017;9(388):eaaj1928. 10.1126/scitranslmed.aaj1928 28469033

[3] Hammitt LL Dagan R Yuan Y Baca Cots M Bosheva M Madhi SA Nirsevimab for prevention of RSV in healthy late-preterm and term infants. N Engl J Med. 2022;386(9):837-46. 10.1056/NEJMoa2110275 35235726

[4] European Medicines Agency (EMA). Beyfortus. Amsterdam: EMA; 2022. Available from: https://www.ema.europa.eu/en/medicines/human/EPAR/beyfortus

[5] RSVAB WGS and GF protocols. Majadahonda: Instituto de Salud Carlos III; 2023. 10.17504/protocols.io.kqdg3xbzqg25/v1

[6] Ewels PA Peltzer A Fillinger S Patel H Alneberg J Wilm A The nf-core framework for community-curated bioinformatics pipelines. Nat Biotechnol. 2020;38(3):276-8. 10.1038/s41587-020-0439-x 32055031

[7] Price MN Dehal PS Arkin AP. FastTree 2--approximately maximum-likelihood trees for large alignments. PLoS One. 2010;5(3):e9490. 10.1371/journal.pone.0009490 20224823 PMC2835736

[8] Garcia-Garcia ML Calvo C Ruiz S Pozo F Del Pozo V Remedios L Role of viral coinfections in asthma development. PLoS One. 2017;12(12):e0189083. 10.1371/journal.pone.0189083 29206851 PMC5716580

[9] Goya S Galiano M Nauwelaers I Trento A Openshaw PJ Mistchenko AS Toward unified molecular surveillance of RSV: A proposal for genotype definition. Influenza Other Respir Viruses. 2020;14(3):274-85. 10.1111/irv.12715 32022426 PMC7182609

[10] Ramaekers K Rector A Cuypers L Lemey P Keyaerts E Van Ranst M. Towards a unified classification for human respiratory syncytial virus genotypes. Virus Evol. 2020;6(2):veaa052. 10.1093/ve/veaa052 33072402 PMC7552823

[11] Teirlinck AC Broberg EK Stuwitz Berg A Campbell H Reeves RM Carnahan A Recommendations for respiratory syncytial virus surveillance at the national level. Eur Respir J. 2021;58(3):2003766. 10.1183/13993003.03766-2020 33888523 PMC8485062

[12] Malboeuf CM Yang X Charlebois P Qu J Berlin AM Casali M Complete viral RNA genome sequencing of ultra-low copy samples by sequence-independent amplification. Nucleic Acids Res. 2013;41(1):e13. 10.1093/nar/gks794 22962364 PMC3592391

[13] Goya S Valinotto LE Tittarelli E Rojo GL Nabaes Jodar MS Greninger AL An optimized methodology for whole genome sequencing of RNA respiratory viruses from nasopharyngeal aspirates. PLoS One. 2018;13(6):e0199714. 10.1371/journal.pone.0199714 29940028 PMC6016902

[14] Graf EH Simmon KE Tardif KD Hymas W Flygare S Eilbeck K Unbiased detection of respiratory viruses by use of RNA Sequencing-based metagenomics: a systematic comparison to a commercial PCR panel. J Clin Microbiol. 2016;54(4):1000-7. 10.1128/JCM.03060-15 26818672 PMC4809917

[15] O’Flaherty BM Li Y Tao Y Paden CR Queen K Zhang J Comprehensive viral enrichment enables sensitive respiratory virus genomic identification and analysis by next generation sequencing. Genome Res. 2018;28(6):869-77. 10.1101/gr.226316.117 29703817 PMC5991510

[16] Agoti CN Otieno JR Munywoki PK Mwihuri AG Cane PA Nokes DJ Local evolutionary patterns of human respiratory syncytial virus derived from whole-genome sequencing. J Virol. 2015;89(7):3444-54. 10.1128/JVI.03391-14 25609811 PMC4403408

[17] Beerenwinkel N Günthard HF Roth V Metzner KJ. Challenges and opportunities in estimating viral genetic diversity from next-generation sequencing data. Front Microbiol. 2012;3:329. 10.3389/fmicb.2012.00329 22973268 PMC3438994

[18] Holmes EC. Error thresholds and the constraints to RNA virus evolution. Trends Microbiol. 2003;11(12):543-6. 10.1016/j.tim.2003.10.006 14659685 PMC7172642

[19] Agoti CN Munywoki PK Phan MVT Otieno JR Kamau E Bett A Transmission patterns and evolution of respiratory syncytial virus in a community outbreak identified by genomic analysis. Virus Evol. 2017;3(1):vex006. 10.1093/ve/vex006 28458916 PMC5399923

[20] Di Giallonardo F Kok J Fernandez M Carter I Geoghegan JL Dwyer DE Evolution of human respiratory syncytial virus (RSV) over multiple seasons in New South Wales, Australia. Viruses. 2018;10(9):476. 10.3390/v10090476 30200580 PMC6164696

[21] Robertson M Eden JS Levy A Carter I Tulloch RL Cutmore EJ The spatial-temporal dynamics of respiratory syncytial virus infections across the east-west coasts of Australia during 2016-17. Virus Evol. 2021;7(2):veab068. 10.1093/ve/veab068 34532066 PMC8438877

[22] Díez-Fuertes F Iglesias-Caballero M García-Pérez J Monzón S Jiménez P Varona S A Founder effect led early SARS-CoV-2 transmission in Spain. J Virol. 2021;95(3):e01583-20. 10.1128/JVI.01583-20 33127745 PMC7925114

[23] Pérez-Sautu U Wiley MR Iglesias-Caballero M Pozo F Prieto K Chitty JA Target-independent high-throughput sequencing methods provide evidence that already known human viral pathogens play a main role in respiratory infections with unexplained etiology. Emerg Microbes Infect. 2019;8(1):1054-65. 10.1080/22221751.2019.1640587 31335277 PMC6691886

