# Supplementary material for: Genomic characterisation of respiratory syncytial virus: a novel system for whole genome sequencing and full-length G and F gene sequences
Source: Euro Surveill. 2023 Dec 7;28(49):2300637. doi: 10.2807/1560-7917.ES.2023.28.49.2300637 (PMC10831411; doi:10.2807/1560-7917.ES.2023.28.49.2300637)
Supplement: Supplement [file 23-00637_CABALLERO_Supplement.pdf]

This supplementary material is hosted by *Eurosurveillance* as supporting information alongside the article “Genomic characterisation of respiratory syncytial virus: a novel system for whole genome sequencing and full-length G and F gene sequences”, on behalf of the authors, who remain responsible for the accuracy and appropriateness of the content. The same standards for ethics, copyright, attributions and permissions as for the article apply. Supplements are not edited by *Eurosurveillance* and the journal is not responsible for the maintenance of any links or email addresses provided therein.

SM. 1. Sequencing parameters for RSV clinical specimens in the targeted whole-genome sequencing (RSVAB WGS) method. The included a description of the subtype, Ct values obtained in the viral RT-PCR used for detection and subtyping, sequencing/assembly metrics for Illumina sequencing, and GISAID accession number. N/A: not applied, the sequencer were not uploaded to the data base due to the low viral coverage

Seq ID	Subtype	Cycle threshold	Reads virus	Average depth of coverage	% genome coverage	Sequencing reagents	GISAID ID
HRSV/A/Spain/MD-224741/2022	A	14,33	4079086	1905	94	NextSeq	EPI_ISL_18463183
HRSV/A/Spain/MD-224420/2022	A	15,1	1339929	923	93	NextSeq	EPI_ISL_18463185
HRSV/A/Spain/MD-224512/2022	A	16,8	6141752	744	98	NextSeq	EPI_ISL_18463167
HRSV/A/Spain/MD-224199/2022	A	15,75	1479714	461	93	NextSeq	EPI_ISL_18463162
HRSV/A/Spain/MD-192731/2019	A	16,1	108192	330	93	MiSeq	EPI_ISL_18463188
HRSV/A/Spain/MD-190041/2018	A	17,08	3131245	402	98	NextSeq	EPI_ISL_18463190
HRSV/A/Spain/MD-224747/2022	A	17,19	5671738	1237	94	NextSeq	EPI_ISL_18463170
HRSV/A/Spain/MD-224870/2022	A	18,06	4013221	483	93	NextSeq	EPI_ISL_18463181
HRSV/A/Spain/MD-192780/2019	A	20,07	96138	827	92	MiSeq	EPI_ISL_18463177
HRSV/A/Spain/MD-224532/2022	A	20,44	3283955	1483	93	NextSeq	EPI_ISL_18463184
HRSV/A/Spain/MD-224979/2022	A	21,1	4201472	684	93	NextSeq	EPI_ISL_18463165
HRSV/A/Spain/MD-192569/2019	A	22,11	60661	235	92	MiSeq	EPI_ISL_18463174
HRSV/A/Spain/MD-192566/2019	A	22,19	105743	419	92	MiSeq	EPI_ISL_18463178
HRSV/A/Spain/MD-224273/2022	A	24,82	3830104	298	93	NextSeq	EPI_ISL_18463186
HRSV/A/Spain/MD-190018/2019	A	25,06	1029584	416	84	NextSeq	EPI_ISL_18463171
HRSV/A/Spain/MD-192775/2019	A	26,04	75640	718	89	MiSeq	EPI_ISL_18463173
HRSV/B/Spain/MD-220011/2022	B	14,01	1576919	155	94	NextSeq	EPI_ISL_18463187
HRSV/B/Spain/MD-191255/2019	B	15,1	24053	215	97	MiSeq	EPI_ISL_18463189

HRSV/B/Spain/MD-190043/2018	B	15,31	1008076	511	94	NextSeq	EPI_ISL_18463191
HRSV/B/Spain/MD-220471/2022	B	16,39	897973	635	93	NextSeq	EPI_ISL_18463172
HRSV/B/Spain/MD-190037/2018	B	17,84	144557	722	91	NextSeq	EPI_ISL_18463192
HRSV/B/Spain/MD-190055/2018	B	18,03	18478	681	91	MiSeq	EPI_ISL_18463179
HRSV/B/Spain/MD-220289/2021	B	19,33	1270851	525	93	NextSeq	EPI_ISL_18463168
HRSV/B/Spain/MD-190038/2018	B	20,4	561536	438	93	NextSeq	EPI_ISL_18463166
HRSV/B/Spain/MD-190030/2018	B	21,52	2044623	470	95	NextSeq	EPI_ISL_18463169
HRSV/B/Spain/MD-224976/2022	B	22,58	1528920	511	95	NextSeq	EPI_ISL_18463180
HRSV/B/Spain/MD-190021/2018	B	22,97	192909	525	94	NextSeq	EPI_ISL_18463175
HRSV/B/Spain/MD-223807/2022	B	23,2	334884	711	90	NextSeq	EPI_ISL_18463176
HRSV/B/Spain/MD-224877/2022	B	25,45	549725	414	94	NextSeq	EPI_ISL_18463182
HRSV/B/Spain/MD-224510/2022	B	26,36	1108906	100	95	NextSeq	EPI_ISL_18463164
HRSV/B/Spain/GA-224031/2022	B	27,04	114713	116	87	NextSeq	EPI_ISL_18463163
HRSV/B/Spain/MD-224022/2022	B	27,99	31391	11	60	NextSeq	N/A
HRSV/B/Spain/MD-224193/2022	B	29,1	83013	11	65	NextSeq	N/A
HRSV/B/Spain/MD-220013/2022	B	29,4	1234	5	40	NextSeq	N(A

SM.2. Sequence identification, subtype, cycle threshold obtained in the characterization PCR, GISAID number of accessions using the targeted RSVGF method.

Seq ID	Subtype	Cycle threshold	G and F complete	GISAID ID
HRSV/A/Spain/MD-224741/2022	A	14,33	Yes	EPI_ISL_18463183
HRSV/A/Spain/MD-224880/2022	A	14,48	Yes	EPI_ISL_18469715
HRSV/A/Spain/MD-224753/2022	A	14,68	Yes	EPI_ISL_18469716
HRSV/A/Spain/MD-224206/2022	A	15,04	Yes	EPI_ISL_18469676
HRSV/A/Spain/MD-224509/2022	A	15,15	Yes	EPI_ISL_18469707
HRSV/A/Spain/MD-224974/2022	A	15,3	Yes	EPI_ISL_18469655
HRSV/A/Spain/MD-220298/2021	A	15,42	Yes	EPI_ISL_18469662
HRSV/A/Spain/MD-221551/2022	A	15,45	Yes	EPI_ISL_18469664
HRSV/A/Spain/MD-223338/2022	A	15,73	Yes	EPI_ISL_18469667
HRSV/A/Spain/MD-224199/2022	A	15,75	Yes	EPI_ISL_18463162
HRSV/A/Spain/MD-224500/2022	A	15,98	Yes	EPI_ISL_18469696
HRSV/A/Spain/MD-224512/2022	A	16,08	Yes	EPI_ISL_18463167
HRSV/A/Spain/MD-224526/2022	A	16,16	Yes	EPI_ISL_18469711
HRSV/A/Spain/MD-224514/2022	A	16,26	Yes	EPI_ISL_18469708

HRSV/A/Spain/MD-224748/2022	A	16,29	Yes	EPI_ISL_18469656
HRSV/A/Spain/MD-224518/2022	A	16,5	Yes	EPI_ISL_18469709
HRSV/A/Spain/MD-224400/2022	A	16,64	Yes	EPI_ISL_18469700
HRSV/A/Spain/MD-220283/2022	A	17,01	Yes	EPI_ISL_18469661
HRSV/A/Spain/MD-224528/2022	A	17,03	Yes	EPI_ISL_18469713
HRSV/A/Spain/MD-224508/2022	A	17,1	Yes	EPI_ISL_18469706
HRSV/A/Spain/MD-223769/2022	A	17,12	Yes	EPI_ISL_18469668
HRSV/A/Spain/MD-224747/2022	A	17,19	Yes	EPI_ISL_18463170
HRSV/A/Spain/MD-224205/2022	A	17,31	Yes	EPI_ISL_18469675
HRSV/A/Spain/MD-224134/2022	A	17,38	Yes	EPI_ISL_18469669
HRSV/A/Spain/MD-224404/2022	A	17,98	Yes	EPI_ISL_18469701
HRSV/A/Spain/MD-224870/2022	A	18,06	Yes	EPI_ISL_18463181
HRSV/A/Spain/MD-224390/2022	A	18,07	Yes	EPI_ISL_18469691
HRSV/A/Spain/MD-224537/2022	A	18,07	Yes	EPI_ISL_18469657
HRSV/A/Spain/MD-224419/2022	A	18,18	Yes	EPI_ISL_18469704
HRSV/A/Spain/MD-222929/2022	A	18,22	Yes	EPI_ISL_18469666
HRSV/A/Spain/MD-224535/2022	A	18,28	Yes	EPI_ISL_18469717
HRSV/A/Spain/MD-224503/2022	A	18,34	Yes	EPI_ISL_18469697
HRSV/A/Spain/MD-224380/2022	A	18,42	Yes	EPI_ISL_18469687
HRSV/A/Spain/MD-224598/2022	A	18,42	Yes	EPI_ISL_18469714
HRSV/A/Spain/MD-224382/2022	A	18,48	Yes	EPI_ISL_18469688
HRSV/A/Spain/MD-224504/2022	A	18,5	Yes	EPI_ISL_18469698
HRSV/A/Spain/MD-224275/2022	A	18,81	Yes	EPI_ISL_18469679
HRSV/A/Spain/MD-224499/2022	A	18,83	Yes	EPI_ISL_18469695
HRSV/A/Spain/MD-224421/2022	A	18,86	Yes	EPI_ISL_18469705
HRSV/A/Spain/MD-224278/2022	A	18,97	Yes	EPI_ISL_18469681
HRSV/A/Spain/MD-224138/2022	A	19,11	Yes	EPI_ISL_18469670
HRSV/A/Spain/MD-224524/2022	A	19,14	Yes	EPI_ISL_18469710
HRSV/A/Spain/MD-224395/2022	A	19,29	Yes	EPI_ISL_18469699
HRSV/A/Spain/MD-224405/2022	A	19,38	Yes	EPI_ISL_18469702
HRSV/A/Spain/MD-224276/2022	A	19,41	Yes	EPI_ISL_18469680
HRSV/A/Spain/MD-222928/2022	A	19,44	Yes	EPI_ISL_18469665
HRSV/A/Spain/MD-224289/2022	A	19,47	Yes	EPI_ISL_18469685
HRSV/A/Spain/MD-224596/2022	A	19,5	Yes	EPI_ISL_18469720
HRSV/A/Spain/MD-224527/2022	A	19,55	Yes	EPI_ISL_18469712
HRSV/A/Spain/MD-224407/2022	A	19,7	Yes	EPI_ISL_18469703
HRSV/A/Spain/MD-224379/2022	A	19,75	Yes	EPI_ISL_18469686
HRSV/A/Spain/MD-224533/2022	A	19,81	Yes	EPI_ISL_18469658
HRSV/A/Spain/MD-224159/2022	A	19,93	Yes	EPI_ISL_18469673
HRSV/A/Spain/MD-224397/2022	A	20,04	Yes	EPI_ISL_18469659
HRSV/A/Spain/MD-224399/2022	A	20,15	Yes	EPI_ISL_18469718
HRSV/A/Spain/MD-224384/2022	A	20,32	Yes	EPI_ISL_18469690
HRSV/A/Spain/MD-220301/2021	A	20,36	Yes	EPI_ISL_18469663
HRSV/A/Spain/MD-224532/2022	A	20,44	Yes	EPI_ISL_18463184
HRSV/A/Spain/MD-224592/2022	A	20,46	Yes	EPI_ISL_18469660

HRSV/A/Spain/MD-224393/2022	A	20,61	Yes	EPI_ISL_18469693
HRSV/A/Spain/MD-224280/2022	A	20,75	Yes	EPI_ISL_18469683
HRSV/A/Spain/MD-224027/2022	A	20,77	Yes	EPI_ISL_18469654
HRSV/A/Spain/MD-224391/2022	A	20,87	Yes	EPI_ISL_18469692
HRSV/A/Spain/MD-224269/2022	A	20,88	Yes	EPI_ISL_18469677
HRSV/A/Spain/MD-224140/2022	A	21,08	Yes	EPI_ISL_18469671
HRSV/A/Spain/MD-224980/2022	A	22,05	Yes	EPI_ISL_18469719
HRSV/A/Spain/MD-224271/2022	A	22,13	Yes	EPI_ISL_18469678
HRSV/A/Spain/MD-224282/2022	A	22,24	Yes	EPI_ISL_18469684
HRSV/A/Spain/MD-224279/2022	A	22,36	Yes	EPI_ISL_18469682
HRSV/A/Spain/MD-224443/2022	A	22,72	Yes	EPI_ISL_18469694
HRSV/A/Spain/MD-224383/2022	A	24,03	Yes	EPI_ISL_18469689
HRSV/A/Spain/MD-224204/2022	A	24,65	Yes	EPI_ISL_18469674
HRSV/A/Spain/MD-224273/2022	A	24,82	Yes	EPI_ISL_18463186
HRSV/A/Spain/MD-224157/2022	A	25,13	Yes	EPI_ISL_18469672
HRSV/B/Spain/MD-220777/2022	B	12,22	Yes	EPI_ISL_18470178
HRSV/B/Spain/MD-220011/2021	B	14,01	Yes	EPI_ISL_18470179
HRSV/B/Spain/MD-224517/2022	B	15,2	Yes	EPI_ISL_18470180
HRSV/B/Spain/MD-220471/2022	B	16,39	Yes	EPI_ISL_18463172
HRSV/B/Spain/MD-224139/2022	B	16,45	Yes	EPI_ISL_18470181
HRSV/B/Spain/MD-224195/2022	B	16,58	Yes	EPI_ISL_18470182
HRSV/B/Spain/MD-224749/2022	B	16,59	Yes	EPI_ISL_18470183
HRSV/B/Spain/MD-224534/2022	B	16,96	Yes	EPI_ISL_18470184
HRSV/B/Spain/MD-223194/2022	B	17,02	Yes	EPI_ISL_18470185
HRSV/B/Spain/GA-220577/2021	B	17,19	Yes	EPI_ISL_18474127
HRSV/B/Spain/MD-224745/2022	B	17,26	Yes	EPI_ISL_18470186
HRSV/B/Spain/MD-224203/2022	B	17,41	Yes	EPI_ISL_18470187
HRSV/B/Spain/MD-224519/2022	B	17,41	Yes	EPI_ISL_18470188
HRSV/B/Spain/MD-224752/2022	B	18,54	Yes	EPI_ISL_18470189
HRSV/B/Spain/MD-224513/2022	B	18,97	Yes	EPI_ISL_18470190
HRSV/B/Spain/GA-220563/2021	B	19	Yes	EPI_ISL_18470191
HRSV/B/Spain/MD-221800/2022	B	19,24	Yes	EPI_ISL_18470192
HRSV/B/Spain/MD-220289/2021	B	19,33	Yes	EPI_ISL_18463168
HRSV/B/Spain/GA-224026/2022	B	19,36	Yes	EPI_ISL_18470193
HRSV/B/Spain/MD-224388/2022	B	19,48	Yes	EPI_ISL_18470194
HRSV/B/Spain/MD-224357/2022	B	20,27	Yes	EPI_ISL_18470195
HRSV/B/Spain/GA-224028/2022	B	20,59	Yes	EPI_ISL_18470196
HRSV/B/Spain/GA-224020/2022	B	20,92	Yes	EPI_ISL_18470197
HRSV/B/Spain/MD-220294/2022	B	21,27	Yes	EPI_ISL_18470198
HRSV/B/Spain/MD-224976/2022	B	22,58	Yes	EPI_ISL_18463180
HRSV/B/Spain/MD-223807/2022	B	23,2	Yes	EPI_ISL_18463176
HRSV/B/Spain/MD-221150/2022	B	24,89	Yes	EPI_ISL_18470199
HRSV/B/Spain/MD-224877/2022	B	25,45	Yes	EPI_ISL_18463182
HRSV/B/Spain/MD-224510/2022	B	26,36	Yes	EPI_ISL_18463164
HRSV/B/Spain/MD-220284/2022	B	26,73	Yes	EPI_ISL_18470200

HRSV/B/Spain/GA-224031/2022	B	27,04	Yes	EPI_ISL_18463163
HRSV/B/Spain/GA-224022/2022	B	27,99	Yes	EPI_ISL_18470201
HRSV/B/Spain/MD-224193/2022	B	29,1	Yes	EPI_ISL_18470202
HRSV/B/Spain/MD-220013/2021	B	29,4	Yes	EPI_ISL_18470203
